# Can we predict arterial lactate from venous lactate in the emergency department?

**DOI:** 10.1186/cc10866

**Published:** 2012-03-20

**Authors:** A Mikami, S Ohde, G Deshpande, T Mochizuki, N Otani, S Ishimatsu

**Affiliations:** 1 St Luke's International Hospital, Tokyo, Japan; 2 St Luke's Life Science Institute, Tokyo, Japan

## Introduction

Analysis of arterial blood has an important role in the clinical assessment of critically ill patients. Particularly, measured arterial lactate (a-Lac) provides valuable information on peripheral circulatory failure, although it is invasive and frequent measurement is often impractical. The aim of this study is to clarify the relationship between a-Lac and the more easily accessed venous lactate (v-Lac) and to generate a formula to predict a-Lac using v-Lac and other laboratory data.

## Methods

A prospective cohort study was conducted from June to November 2011 in the emergency department at a tertiary-level community hospital in Tokyo, Japan. Patients were eligible for entry into the study if an arterial blood gas (ABG) analysis was required for appropriate diagnostic care by the treating physician. Arterial and venous samples were taken within 5 minutes of each other from the ipsilateral radial artery and cephalic vein. Samples were analyzed as soon as possible after collection on the same blood gas analyzer. Univariate linear regression analysis was conducted to generate an equation to calculate a-Lac incorporating only v-Lac. Then, a multivariate forward stepwise logistic regression model (*P *value of 0.05 for entry, 0.1 for removal) was used to generate an equation including v-Lac and other potentially relevant variables including age, sex, systolic blood pressure, heart rate, and venous blood parameters (pH, pO_2_, pCO_2_, hemoglobin, creatine kinase, potassium). A Bland-Altman plot was drawn and the two equations were compared for model fitting using R-squared.

## Results

Seventy-two arterial samples from 72 patients (61% male; mean age, 58.2 years) were included in the study. Indications for ABG included respiratory failure (16%), assessment of shock (21%), altered mental status (26%), and others (36%). An initial regression equation was derived from univariate linear regression analysis: (a-Lac) = -0.259 + (v-Lac) × 0.996. Subsequent multivariate forward stepwise logistic regression analysis, incorporating venous lactate and venous pO_2 _(v-pO_2_), generated the following equation: (a-Lac) = -0.469 + (v-pO_2_) × 0.005 + (v-Lac) × 0.997. Calculated R-squared values by single and multiple regression were 0.94 and 0.96, respectively.

## Conclusion

Venous lactate estimates showed a high correlation with arterial values and our data provide two clinically useful equations to calculate a-Lac from v-Lac data. Considering clinical flexibility, Lac = -0.259 + VLac × 0.996 might be more useful, while avoiding a time-consuming and invasive procedure.

